# Supplementary material for: Effect of Cancer-Related Cachexia and Associated Changes in Nutritional Status, Inflammatory Status, and Muscle Mass on Immunotherapy Efficacy and Survival in Patients with Advanced Non-Small Cell Lung Cancer
Source: Cancers (Basel). 2023 Feb 8;15(4):1076. doi: 10.3390/cancers15041076 (PMC9953791; doi:10.3390/cancers15041076)
Supplement: Supplementary file 1 [file cancers-15-01076-s001.zip › cancers-2144442-supplementary.pdf]

Supplementary files

Methods S1. Calculation of LBM and SMI based on the equations provided by Mourtzakis et al [39]

LBM/total skeletal muscle cross-sectional area (TMA) (kg) = 0.3 × skeletal muscle area at L3 (TMA) (cm2) + 6.06 (1)

Fat mass (FM) (kg) = 0.042 × total fat tissue area at L3 (cm2) + 11.2 (2)

Subsequently, both parameters were corrected to individual patient height using the following formulas:

Fat-free mass (FFM) index (FFMI; kg/m2) = Total FFM/height squared (m2) (3)

FM index (FMI; kg/m2) = Total FM/height squared (m2) (4)

For the assessment of sarcopenia, we calculated the SMI (which is the cross-sectional area of the bilateral psoas muscles at L3) by dividing LBM/TMA at L3 by the height squared:

SMI (cm2/m2) = TMA at L3/height squared (m2) (5)

Figure S1

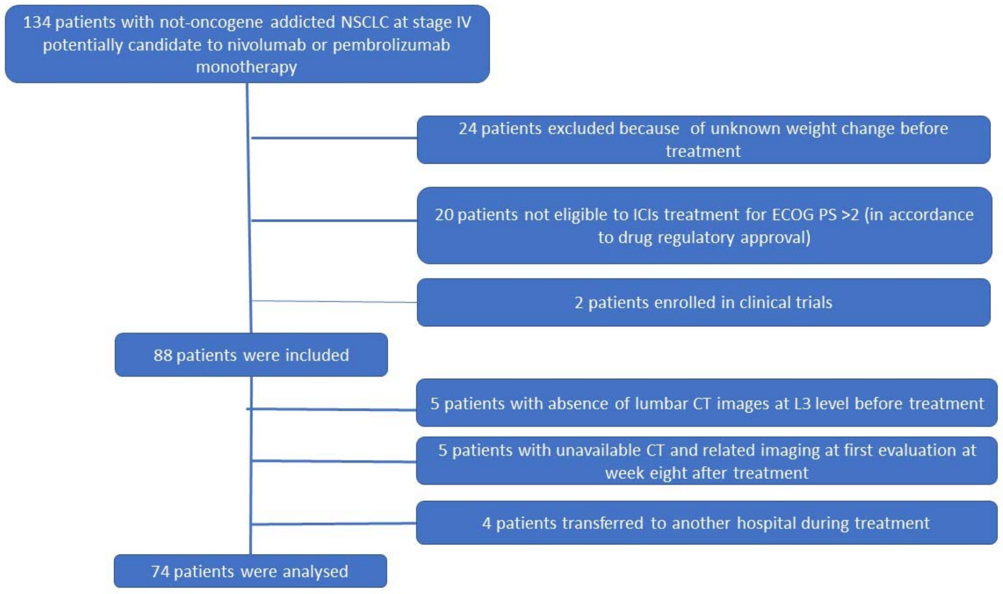

Figure S1. Flow diagram of the study. Abbreviations: NSCLC, non-small-cell lung cancer; ECOG PS, Eastern Cooperative Oncology Group Performance Status; CT, computed tomography

Table S1. Analysis of miniCASCO Score and its subscales at baseline.

Parameters	Mean±SD (range)
miniCASCO	32.5 ± 16.4 (6-73)
miniCASCO subscales	
BWC	8.3 ± 2 (0-40)
IMD	7.9 ± 2.2 (0-16)
PHP	8.3 ± 4.9 (0-15)
Anorexia	4.9 ± 2.9 (0-20)
QoL	4.1 ± 1.7 (2-10)

Data are reported as mean ±standard deviation (SD). Abbreviations: BWC, bodyweight composition/change; IMD, inflammation/metabolic disturbances/immunosuppression; PHP, physical performance (PHP); QoL, quality of life.

Table S2. Univariate COX-regression analysis of variables determinants of PFS.

PFS			
Variables	b coefficient	Exp (b); 95%CI	p value
Baseline weight loss %	0.01218	1.0123 (0.9544 to 1.0736)	0.6849
Cachexia according to Fearon criteria	0.3008	1.3509 (0.7773 to 2.3480)	0.2861
LBM	0.01933	1.0195 (0.9876 to 1.0525)	0.2338
SMI	0.01921	1.0194 (0.9971 to 1.0422)	0.0885
C-reactive protein	0.00252	1.0025 (0.9964 to 1.0086)	0.4165
IL-6	0.03155	1.0321 (1.0176 to 1.0468)	**<0.0001**
Neutrophils count	0.0000071	1.0000 (0.9999 to 1.0001)	0.8996
Lymphocyte count	0.000406	1.0004 (0.9999 to 1.0009)	0.1222
NLR	-0.03765	0.9630 (0.8473 to 1.0946)	0.5644
Albumin	-0.06222	0.9397 (0.4607 to 1.9166)	0.8642
mGPS	0.4079	1.5037 (1.1087 to 2.0396)	**0.0087**
Hemoglobin	-0.07542	0.9274 (0.8045 to 1.0690)	0.2983
MiniCASCO score	0.07253	1.0752 (1.0137 to 1.1405)	**0.0159**
Anorexia subscale	-0.01862	0.9816 (0.9330 to 1.0327)	0.4722
IMD subscale	0.09010	1.0943 (1.0277 to 1.1652)	**0.0049**
BWC subscale	0.00326	1.0033 (0.9800 to 1.0271)	0.7854
PHP subscale	0.01395	1.0140 (0.9983 to 1.0330)	0.0807
QoL subscale	0.1862	1.2046 (1.0516 to 1.3799)	**0.0072**
MiniCASCO-based cachexia severity	0.2510	1.2854 (1.1244 to 1.7075)	**0.0083**

Significant p values (<0.05) are reported in bold. Abbreviations: PFS, Progression-Free Survival; LBM, lean body mass; SMI, skeletal mass index; IL, Interleukin; NLR, neutrophil-to-lymphocyte ratio; mGPS, modified Glasgow Prognostic Score; BWC, bodyweight composition/change; IMD, inflammation/metabolic disturbances/immunosuppression; PHP, physical per-formance (PHP); QoL, quality of life.

Table S3. Univariate COX-regression analysis of variables determinants of OS.

OS			
**Variables**	**b coefficient**	**Exp (b) (95%CI)**	**p value**
Baseline weight loss %	0.04044	1.0413 (0.9802 to 1.1062)	0.1897
Cachexia according to Fearon criteria	0.1306	1.1396 (1.4632 to 1.8875)	**0.0306**
LBM	0.01849	1.0187 (0.9847 to 1.0538)	0.2852
SMI	0.02162	1.0219 (0.9967 to 1.0477)	0.0895
C-reactive protein	0.004677	1.0047 (0.9984 to 1.0110)	0.1412
IL-6	0.03825	1.0390 (1.0213 to 1.0570)	**<0.0001**
Neutrophils count	0.00007124	1.0001 (1.000 to 1.0002)	0.2054
Lymphocyte count	0.0002870	1.0003 (0.9998 to 1.0008)	0.2617
NLR	0.01045	1.0105 (0.8892 to 1.1484)	0.8728
Albumin	-0.03547	0.9652 (0.4602 to 2.0243)	0.9252
mGPS	0.4847	1.6237 (1.1771 to 2.2396)	**0.0031**
Hemoglobin	-0.06254	0.9394 (0.8082 to 1.0919)	0.9394
MiniCASCO score	0.1619	1.0163 (1.0341 to 1.9989)	**0.0047**
Anorexia subscale	-0.000003	1.000 (0.9502 to 1.0524)	0.9999
IMD subscale	0.1021	1.1075 (1.0300 to 1.1908)	**0.0058**
BWC subscale	0.009881	1.0099 (0.9845 to 1.0360)	0.4472
PHP subscale	0.05954	1.0613 (0.9944 to 1.1328)	0.0732
QoL subscale	0.1287	1.1374 (0.9658 to 1.3395)	0.1229
MiniCASCO-based cachexia severity	0.6540	1.9233 (1.0252 to 3.6080)	**0.0416**

Significant p values (<0.05) are reported in bold. Abbreviations: OS, Overall Survival; LBM, lean body mass; SMI, skeletal mass index; IL, Interleukin; NLR, neutrophil-to-lymphocyte ratio; mGPS, modified Glasgow Prognostic Score; BWC, bodyweight composition/change; IMD, inflammation/metabolic disturbances/immunosuppression; PHP, physical performance (PHP); QoL, quality of life.

RESULTS S1

Association of cachexia status, miniCASCO, body composition, inflammatory status, and nutritional status with survival outcomes

The correlation analysis demonstrated a statistically significant inverse correlation between PFS and CRP level (r= -0.3333; 95% CI, -0.529 to -0.103; *p*=0.0055), IL-6 level (r= -0.649; 95% CI, -0.775 to -0.472; *p*<0.0001), and miniCASCO (r= -0.210; 95% CI, -0.3679 to -0.0019; *p*=0.0418). Regarding miniCASCO subscale scores, PFS elicited an inverse significant correlation with IMD (r= -0.324; 95% CI, -0.522 to -0.0932; *p*=0.0070) and QoL (r= -0.309; 95% CI, -0.510 to-0.0761; *p*=0.0104) subscale scores. Results of correlation analysis are reported in Table S4.

Table S4. Correlation analysis between parameters of cachexia status, body composition/nutritional status, inflammatory status, and miniCASCO with survival outcomes (PFS and OS).

	**PFS**		**OS**	
**Parameters**	**r coefficient (CI 95%)**	**p value**	**r coefficient (CI 95%)**	**p value**
Baseline weight loss %	-0.0996 (-0.334 to 0.146	0.4262	-0.165 (-0.392 to 0.0798)	0.1843
LBM	0.150 (-0.2060 to 0.04952)	0.226	0.250 (0.2727 to 0.004086)	**0.0436**
SMI	0.0917 (0.323 to -0.150)	0.4571	0.275 (0.481 to 0.0387)	**0.0235**
C-reactive protein	-0.333 (-0.529 to -0.103)	**0.0055**	-0.388 (-0.574 to -0.165)	**0.0011**
IL-6	-0.649 (-0.775 to -0.472)	**<0.0001**	-0.628 (-0.760 to -0.444)	**<0.0001**
Neutrophils count	-0.222 (-0.437 to 0.0172)	0.0686	-0.346 (-0.540 to -0.117)	**0.0039**
Lymphocyte count	0.210 (-14.6686 to 0.9789)	0.085	0.250 (0.4288 to 0.02784)	**0.0446**

NLR	0.0692 (-0.172 to 0.303)	0.5748	-0.0913 (-0.323 to 0.150)	0.4591
Albumin	0.0597 (-0.181 to 0.294)	0.6287	0.0614 (-0.180 to 0.295)	0.6190
Hemoglobin	0.116 (-0.126 to 0.345)	0.3458	0.152 (-0.0895 to 0.377)	0.2153
MiniCASCO score	-0.210 (-0.3679 to -0.0119)	**0.0418**	-0.252 (-0.463 to -0.0144)	**0.0382**
Anorexia subscale	0.185 (-0.0559 to 0.406)	0.1309	0.253 (0.0157 to 0.464)	**0.0372**
IMD subscale	-0.324 (-0.522 to -0.0932)	**0.0070**	-0.462 (-0.631 to -0.251)	**0.0001**
BWC subscale	-0.145 (-0.371 to 0.0969)	0.2384	-0.131 (-0.358 to 0.111)	0.2868
PHP subscale	-0.0931 (-0.324 to 0.149)	0.4503	-0.182 (-0.403 to 0.0586)	0.1366
QoL subscale	-0.309 (-0.510 to -0.0761)	**0.0104**	-0.352 (-0.545 to -0.124)	**0.0032**

Significant p values (<0.05) are reported in bold. Abbreviations: LBM, lean body mass, SMI, skeletal mass index; IL, Interleukin; NLR, neutrophil-to-lymphocyte ratio; BWC, bodyweight composition/change; IMD, inflammation/metabolic disturbances/immunosuppression; PHP, physical per-formance (PHP); QoL, quality of life.

Among categorical variables, we found a significant difference in PFS between mGPS (*p*=0.005) and miniCASCO-based cachexia severity severity (*p*<0.001) categories (Figure S2).

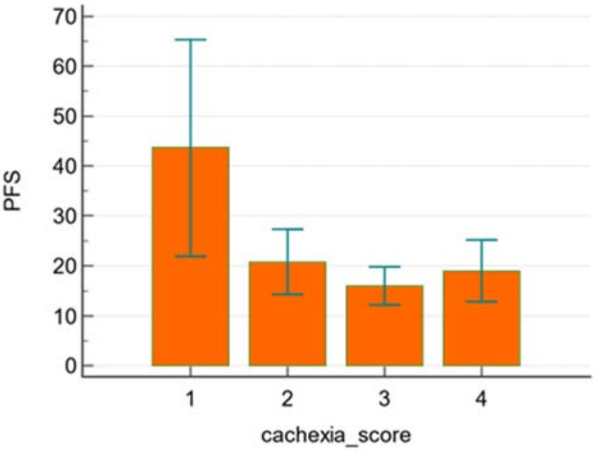

Figure S2. Comparison of progression-free survival according to cachexia severity. Progression-free survival (PFS) was significantly different among patients with different categories of cachexia severity assessed by miniCASCO. Groups were compared by ANOVA test (p<0.001).

Furthermore, we found a statistically significant inverse correlation of OS with CRP level (r= -0.388; 95% CI, -0.574 to -0.165; *p*=0.0011), IL-6 level (r= -0.628; 95% CI, -0.760 to -0.444; *p*<0.0001), neutrophil absolute count (r= -0.346; 95% CI, -0.540 to -0.117; *p*=0.0039), mGPS (r= -0.364; 95% CI, -0.555 to -0.138; *p*=0.0023), miniCASCO score (r= -0.252; 95% CI, -0.463 to -0.0144; *p*= 0.0382), and miniCASCO subscale scores (IMD [r= -0.462; 95% CI, -0.631 to -0.251; *p*=0.0001] and QoL [r= -0.352; 95% CI, -0.545 to -0.124; *p*=0.032]). A positive significant correlation was found between OS and LBM (r=0.250; 95% CI, 0.2727 to 0.004086; *p*=0.0436), SMI (r=0.275; 95% CI, 0.481 to 0.0387; *p*=0.0235), lymphocyte absolute count (r= 0.250; 95% CI 0.4288 to 0.02784; *p*=0.0446), and

anorexia subscale score (r=0.253; 95% CI, 0.0157 to0.464; *p*=0.0372). Results of correlation analysis are reported in Supplementary Table S4.

Among categorical variables we found a significant difference (by Krusakll-Wallis test) in OS in terms of mGPS (*p*=0.0107), the presence of sarcopenia (*p*=0.0197), and miniCASCO-based cachexia severity (*p*=0.023) (Figure S3).

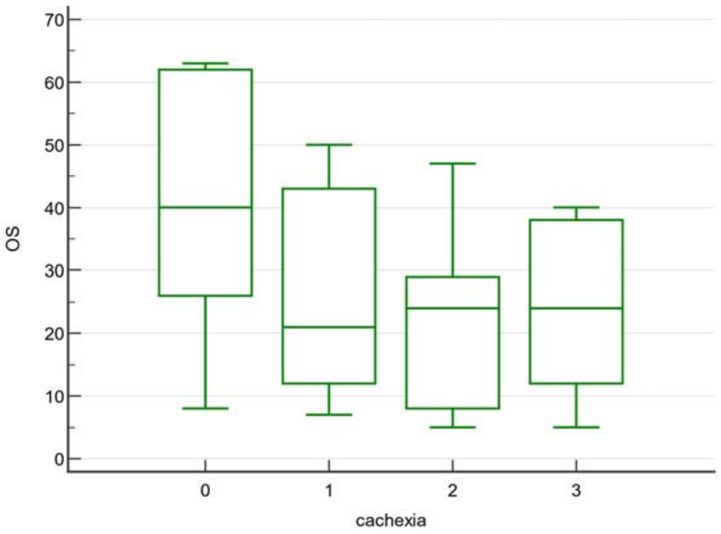

Figure S3. Comparison of overall survival according to cachexia severity. Overall survival (OS) was significantly different among patients with different categories of cachexia severity assessed by miniCASCO. Groups were compared by Kruskall-Wallis test (p=0.023).

Figure S4.

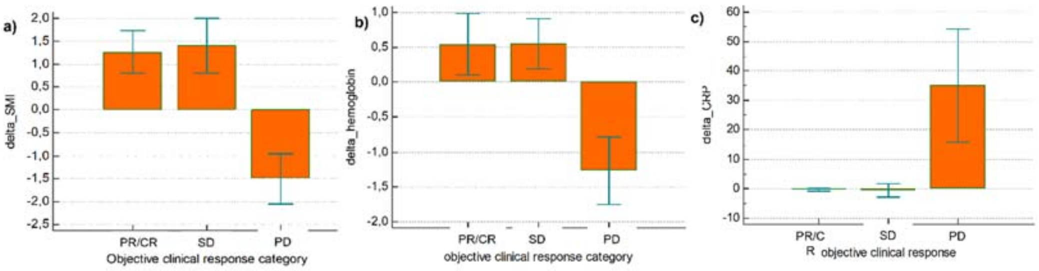

Figure S4. Changes of skeletal muscle index (SMI), hemoglobin and C-reactive Protein (CRP) values during treatment according to objective clinical response categories. a) patients with progression of disease (PD) had a significantly different decrease in SMI in comparison to patients with partial response (PR)/complete response (CR) and stable disease (SD) (p=0.003); b) patients with PD had a significantly different decrease in hemoglobin than patients with PR/CR and SD (p=0.011); c) patients with PD had a significantly different increase in C-reactive protein (CRP) levels in comparison to patients with PR/CR and SD (p=0.008). Differences between categories were calculated by ANOVA test.
